# Systematically variable planktonic carbon metabolism along a land-to-lake gradient in a Great Lakes coastal zone

**DOI:** 10.1093/plankt/fbu066

**Published:** 2014-08-11

**Authors:** Anthony D. Weinke, Scott T. Kendall, Daniel J. Kroll, Eric A. Strickler, Maggie E. Weinert, Thomas M. Holcomb, Angela A. Defore, Deborah K. Dila, Michael J. Snider, Leon C. Gereaux, Bopaiah A. Biddanda

**Affiliations:** 1 Annis Water Resources Institute, Grand Valley State University, 740 West Shoreline Drive, Muskegon, MI 49441, USA; 2 Munson Medical Center, 1105 6th Street, Traverse City, MI 49684, USA; 3 Salish Sea Expeditions, 647 Horizon View Pl Nw, Bainbridge Isle, WA 98110, USA; 4 Herman Miller, 855 East Main Street, Mail Stop 0156, Zeeland, MI 49464, USA; 5 USC Baruch Marine Field Lab, U. South Carolina, Po Box 1630, Georgetown, SC 29442, USA; 6 School of Freshwater Sciences, U. Wisconsin-Milwaukee, 600 E. Greenfield Ave, Milwaukee, WI 53204, USA

**Keywords:** coastal, pelagic, production, respiration, gradient

## Abstract

During the summers of 2002–2013, we measured rates of carbon metabolism in surface waters of six sites across a land-to-lake gradient from the upstream end of drowned river-mouth Muskegon Lake (ML) (freshwater estuary) to 19 km offshore in Lake Michigan (LM) (a Great Lake). Despite considerable inter-year variability, the average rates of gross production (GP), respiration (R) and net production (NP) across ML (604 ± 58, 222 ± 22 and 381 ± 52 µg C L^−1^ day^−1^, respectively) decreased steeply in the furthest offshore LM site (22 ± 3, 55 ± 17 and −33 ± 15 µg C L^−1^day^−1^, respectively). Along this land-to-lake gradient, GP decreased by 96 ± 1%, whereas R only decreased by 75 ± 9%, variably influencing the carbon balance along this coastal zone. All ML sites were consistently net autotrophic (mean GP:R = 2.7), while the furthest offshore LM site was net heterotrophic (mean GP:R = 0.4). Our study suggests that pelagic waters of this Great Lakes coastal estuary are net carbon sinks that transition into net carbon sources offshore. Reactive and dynamic estuarine coastal zones everywhere may contribute similarly to regional and global carbon cycles.

## INTRODUCTION

The exchange of carbon between atmospheric, terrestrial and aquatic pools is one of the largest biogeochemical pathways on Earth, second only to the water cycle (Schlesinger and Berhardt, 2013). The dual processes of autotrophy and heterotrophy drive the biosphere's carbon cycle, making their quantification essential for estimating the magnitude of carbon flux and quantifying the net carbon balance of ecosystems. However, measurements that enable such assessments, coupled measurements of photosynthesis and respiration, are severely lacking in aquatic ecosystems (del Giorgio *et al*., 1997; Karl *et al*., 2003; Serret *et al*., 2001).

Carbon and nutrients that run off or drain through the terrestrial environments of watersheds pass through the myriad of waterways consisting of streams, rivers, wetlands and lakes on to receiving basins such as the Great Lakes and the oceans (Beman *et al*., 2005; Biddanda and Cotner, 2002). However, inland waters do not play a passive role as mere conduits of carbon and nutrients in this worldwide phenomenon. Indeed, recent studies have shown that inland waters of the world, representing only 1% of the Earth's surface area, play a disproportionately large role in the global carbon cycle (Cole *et al*., 2007; Tranvik *et al*., 2009). In particular, significant processing of terrigenous nutrients and carbon occurs in land-margin ecosystems such as streams, rivers, estuaries and coastal zones (Marko *et al*., 2013). It is estimated that annually of the about 5 Pg of carbon entering the inland waters, ∼0.2–0.6 Pg of carbon is buried in freshwater sediments (twice the annual burial into oceanic sediments), and ∼0.7–4.0 Pg of carbon is respired to the air in freshwater systems (equal to the net uptake of carbon by the oceans). These estimates lend credence to the emerging notion that inland waters are highly reactive and play a crucial and hitherto underappreciated role in regional and global carbon cycles (Raymond *et al*., 2013; Wehrli, 2013).

The North American Great Lakes contain nearly 20% of Earth's liquid surface freshwater. Though large, they are heavily human-impacted systems with stressors acting on multiple fronts (Cuhel and Aguilar, 2013; Evans *et al*., 2011; Rothlisberger *et al*., 2010). Climate change, for example, is resulting in warmer temperatures occurring earlier in the year leading to earlier spring blooms followed by a cascade of ecosystem effects (Russ *et al*., 2004). Furthermore, in recent years, invasive dreissenid mussels have nearly eliminated the winter and spring blooms (Kerfoot *et al*., 2010; Vanderploeg *et al*., 2010). Studies suggest that the annual stream and river discharge into southern Lake Michigan (LM), equaling ∼1% of its total volume, have terrestrial subsidies of dissolved organic carbon (DOC) and phosphorus 10 and 20 times higher than LM, that support up to 20% of the phytoplankton production and up to 10% of heterotrophic bacterial production in southern LM (Biddanda and Cotner, 2002). LM is currently on its way to becoming as oligotrophic as Lake Superior (Evans *et al*., 2011; Mida *et al*., 2010), making terrestrial subsidies of nutrients and organic matter more vital than ever to its annual balance (Johengen *et al*., 2008).

River-mouth estuaries in particular can be biogeochemical hotspots where the land interacts with the water (McClain *et al*., 2003). As incoming water slows in these estuarine environments, the increased residence time enables suspended particles to settle and allows plankton more time to assimilate nutrients and carbon (Essington and Carpenter, 2000; Slattery and Phillips, 2011). These processes influence the river's export of inorganic nutrients and organic matter to receiving basins such as near-shore LM (Marko *et al*., 2013). Carbon assimilated as biomass by primary producers supports heterotrophic secondary production, as well as the burial of excess carbon in the sediments. River-mouth estuaries capture the influence of entire watersheds, thus human activities and climate change can have a heavy impact at such land-water transition zones (Allan *et al*., 2012; Smith and Hollibaugh, 1993).

**Fig.1.**
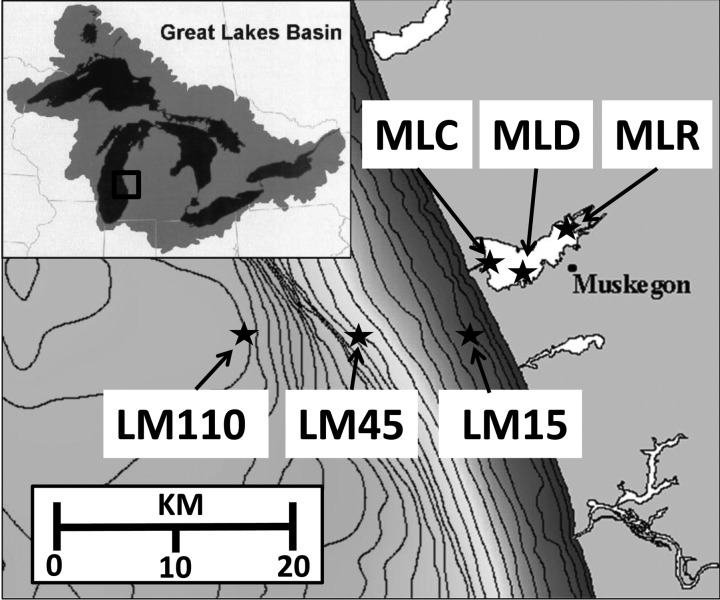
Map of Muskegon Lake and coastal Lake Michigan showing sample sites in Muskegon Lake (MLR, Muskegon River; MLD, Muskegon Lake Deep Hole; MLC, Muskegon Channel) and Lake Michigan (LM15, Lake Michigan 15 m isobath; LM45, Lake Michigan 45 m isobath; LM110, Lake Michigan 110 m isobaths).

A good example of an “inland” lake with connectivity to a larger drainage basin is Muskegon Lake (ML), a drowned river-mouth lake on Michigan's western coast (Steinman *et al*., 2008). ML is a mesotrophic drowned river-mouth lake (16.8 km^2^), fed by the second largest watershed in Michigan (6500 km^2^) which flows into LM's eastern shore through a channel (Marko *et al*., 2013; Steinman *et al*., 2008; Fig.1). This estuarine lake directly connects the Muskegon River and its watershed to LM, the second largest North American Great Lake (Larson *et al*., 2013; Marko *et al*., 2013). The ∼1700 km length of the North American Great Lakes' shoreline is intersected by almost 3000 tributaries that have significant economic and ecological importance (Larson *et al*., 2013; Marko *et al*., 2013).

The objective of this study, starting in 2002, was to monitor the rates of carbon metabolism in surface waters during the summer at six sites from ML out into LM. We tracked the changes in pelagic gross primary production (GP), respiration (R) and net primary production (NP) from year to year, as well as site-specific differences. Our goal was to quantify differences in rates of GP, R and NP as water flows further from the land, and to test the emerging hypothesis that along this land-to-lake gradient, nearshore autotrophy is gradually giving way to offshore heterotrophy in response to changing environmental variables (e.g. temperature) over the last 11 years.

## METHOD

### Sampling locations

Our study sites include three locations in ML (upstream, center, downstream) and three locations in LM that extend from nearshore out to 19 km offshore (Fig.1). The location names, depths and positions for ML are MLR (8 m: 43 15.008N, 86 15.342W) upstream near the river-mouth, MLD (25 m: 43 14.006N, 86 19.389W) located over ML's deepest spot and MLC (10 m, 43 13.397N, 86 17.884W) located near the Muskegon channel opening. LM sample locations follow an east-west transect near the Muskegon harbor and include LM15 (15 m: 43 11.28N, 86 20.64W), LM45 (45 m: 43 11.281N, 86 25.92W) and LM110 (43 11.28N, 86 32.16W).

### Field sample collection and metabolism measurements

Surface water was collected using a tethered bucket and combined into 20 L acid-cleaned carboys once during the summers of 2002–2013 within the period of mid-July to early-August. ML sites were sampled on a different day than the LM sites, but both lakes were sampled within 3 weeks of each other. Water was collected in the early morning when possible (or late night). Carboys were kept on ice in coolers and transported to the lab in less than 2 h for processing at Annis Water Resources Institute (AWRI) in Muskegon, Michigan. Water was dispensed into quadruplicate initial, light and dark BOD bottles, and then incubated for 24 h (48 h in the case of LM respiration 2002–2004) suspended under similar *in situ* temperature and light conditions in ML off the AWRI pier at 1 m depth (Biddanda *et al*., 2008).

NP and R estimates were obtained from changes in dissolved oxygen in light and dark incubations, respectively. Winkler titrations for the determination of dissolved oxygen were carried out directly in 300 mL BOD bottles using a Radiometer TitraLab(R) 860 automatic titrator with potentiometric endpoint detection using a combined platinum Ag/AgCl reference electrode (Biddanda *et al*., 2001; Granéli and Graneli, 1991). Data from a minimum of three replicates were used for calculations, with the most outlying fourth replicate removed from the data set resulting in significantly reduced percent coefficient of variation (*P* < 0.05) (Carignan *et al*., 1998). GP was calculated from measured NP and R using the formula GP = NP + R. Any negative GP and/or R values most likely represent an error at some step in the experiments, and all such associated GP and R data were removed from the data set (Biddanda *et al*., 2008; Pomeroy *et al*., 1994). We evaluated the differences among the rates of GP, R and NP by using a *t*-test to compare slopes of their linear regressions.

### Additional data acquisition and analyses

An analysis of multiple parameters that could act as drivers of metabolism was performed on: chlorophyll *a* (chl *a*) concentrations, surface water temperature, total precipitation, river discharge and daily average photosynthetically active radiation (PAR). With temperature, for example, GP, R and NP were plotted against their corresponding water temperatures to find how metabolism reacted to varying water temperature. Associated *r*^2^ values were calculated for the best appropriate relationship attainable from trial plots, linear, exponential or polynomial regressions.

Chl *a* concentrations at the various sites were determined through a variety of methods. As measurement of chl *a* was not part of the original study, available data were gathered at the time of the final analysis. So the years and sites mentioned below were the only periods for which chl *a* data were available. The 2006 chl *a* value was taken with a Hydrolab (R) (OTT Hydromet) directly at the sample site from the water surface. The 2010 summer surface chl *a* data for LM were obtained from sensors towed behind vessels (H. Vanderploeg personal communication, NOAA). 2011–2013 ML chl *a* concentrations were obtained from the ML Observatory (MLO; www.gvsu.edu/buoy). The chl *a* data were averaged from the MLO's 2 m sensor for the 24-h period in which the BOD bottle incubations were performed. In 2013, LM chl *a* concentrations were measured by inserting a YSI (TM) sonde (YSI Incorporated) equipped with a chl *a* sensor into the surface water samples from each of the three LM sites.

The relationship of GP, R and NP to chl *a* was analyzed two ways. The first was via linear regressions of increasing metabolism and chl *a* in ML, LM and the lakes combined. The averages of chl *a*, GP, R and NP were also compared among ML and LM via *t*-test. This allowed not only to study how the rates of GP, R and NP change with increasing chl *a*, but also how the two lakes differed with respect to chl *a*.

Water temperature data were taken at the site when the water was sampled. Surface water temperatures were plotted along with determined GP, R and NP values to see how metabolism varied with temperature. All temperature and metabolism data were combined and each metabolism parameter was plotted against their corresponding temperatures. Exponential regression values were determined for each parameter, based on previous finding of an exponential relationship between phytoplankton growth rate and temperature (Eppley, 1972).

The precipitation data are monthly totals taken from the weather underground archive for Muskegon, MI (www.weatherunderground.com). Precipitation analysis was performed by plotting GP, R and NP against the previous 3 months total of precipitation, and GP, R and NP per unit precipitation were all plotted against precipitation.

Total daily discharge for periods preceding the sampling date for the Muskegon River from the USGS gage at the Croton Dam (75 km upstream) in Croton, MI, was obtained from the USGS website (http://waterdata.usgs.gov/nwis). A more rigorous analysis was performed to see if total preceding discharge had any relation to variation in GP, R or NP, or if there was a particular time period before sampling that could also explain variation (discharge 15, 30, 45, 60, 75 and 90 days before sampling lag period). GP, R and NP were plotted against these two types of discharge totals.

PAR data were obtained from the NOAA GLERL Real-Time Meteorological Observatory Network met station at the LM field station in Muskegon, MI (http://www.glerl.noaa.gov/metdata/mkg/). The PAR values used to represent the average of the 5-min interval time-series data for the day of incubation. Again, GP, R and NP were plotted against daily average PAR. GP, R and NP per unit PAR were plotted against PAR as well.

## RESULTS

**Fig.2.**
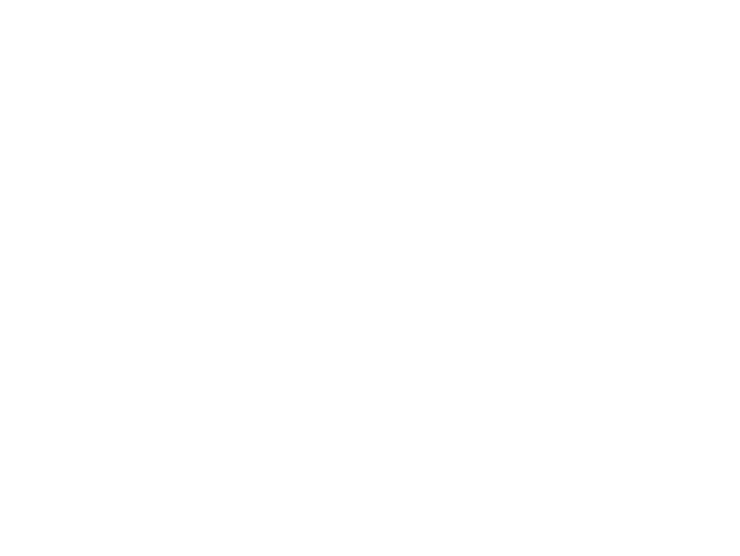
Average GP, R and NP for surface water samples collected during each summer from 2003–2013 at six sample sites from Muskegon River to the Lake Michigan 110 m isobath (error bars represent 1 standard error). Letters (**A**, **B**, or **C**) shown above the bars show metabolic parameters that are statistically different, (*P* < 0.05 in *t*-test) and shown in the order of GP–R–NP. Number of samples (*n*) are also shown above the bars.

**Fig.3.**
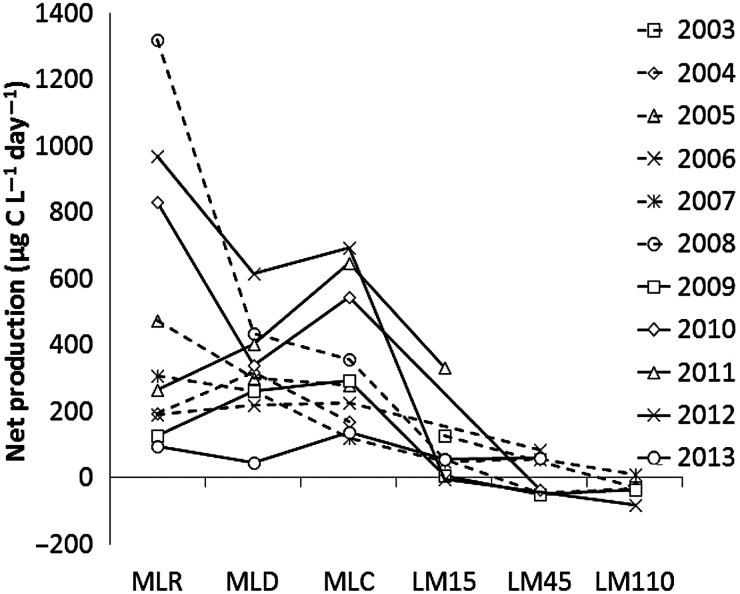
Year-by-year trend in surface water NP at each of the six sites from 2003 to 2013 showing that NP systematically decreases from Muskegon Lake to Lake Michigan, even though the decrease is not a constant and some years are more productive and some less productive.

The average rates of pelagic GP, R, NP and the ratio of GP:R along the land-to-lake gradient decreased with increasing distance from Muskegon River (Table I, Fig.2). The rates of GP, R and NP were all found to be significantly different from one another along this land-to-lake gradient based on a comparison of the slopes of the regressions (*P*-value < 0.05). In general, GP decreased at a faster rate than *R*, thus the average NP that was positive in ML decreased to nearly zero out at LM45 in LM, and was negative most of the years at LM110 (Table I, Fig.3). Along the transect, average NP (in units of µg C L^−1^day^−1^) was 478 for MLR, 320 for MLD, 347 for MLC, 90 for LM15, 12 for LM45 and −33 for LM110. Using the average GP and R rates, corresponding GP:*R* ratios were calculated to be 2.9 for MLR, 2.5 for MLD, 2.7 for MLC, 2.1 for LM15, 1.2 for LM45 and 0.4 for LM110.

**Table I:** GP, R and NP values (μg C L^−1^day^−1^) measured in surface waters during the summer along the land-to-lake gradient during 2002–2013

	MLR			MLD			MLC			LM15			LM45			LM110		
Year	GP	R	NP	GP	R	NP	GP	R	NP	GP	R	NP	GP	R	NP	GP	R	NP
2002	NM	NM	NM	NM	NM	NM	NM	NM	NM	NM	18	NM	NM	260	NM	NM	NM	NM
2003	NM	NM	NM	NM	NM	NM	NM	NM	NM	144	16	128	NM	36	NM	16	45	−28
2004	292	96	196	402	83	319	222	52	170	NM	63	NM	NM	NM	NM	NM	20.4	NM
2005	699	225	474	441	142	299	424	146	278	NM	40	NM	NM	NM	NM	NM	NM	NM
2006	296	104	192	319	100	219	364	139	226	ED	ED	ED	117	32	85	ED	ED	ED
2007	682	376	306	480	220	261	295	173	121	137	88	49	76	21	55	18	5	12
2008	1653	334	1319	610	175	435	472	114	358	183	128	55	25	70	−45	22	54	−32
2009	286	160	126	491	231	260	498	206	292	80	74	6	48	99	−51	23	58	−35
2010	1151	318	833	605	265	340	856	310	546	NM	NM	NM	46	82	−36	NM	NM	NM
2011	452	186	266	595	193	402	892	244	648	424	90	334	NM	NM	NM	ED	ED	ED
2012	1200	230	971	776	159	617	881	189	692	126	128	−2	ED	ED	ED	32	112	−80
2013	561	467	94	590	544	46	625	487	138	93	36	57	69	8	61	ED	ED	ED
Average pairs	727	250	478	531	211	320	553	206	347	169	80	90	64	52	12	22	55	−33
Std error pairs	147	38	133	41	41	47	79	38	66	44	16	44	13	15	25	3	17	15
P:R pairs	2.9			2.5			2.7			2.1			1.2			0.4		
Slope	41	21	20	34	30	5	68	32	36	4	6	0	−5	−12	−3	2	10	−6
*r*^2^	0.07	0.27	0.02	0.64	0.48	0.01	0.68	0.62	0.27	0.01	0.12	0.0	0.13	0.01	0.02	0.85	0.44	0.37
*P*-value	0.46	0.12	0.69	<0.05	<0.05	0.8	<0.05	<0.05	0.13	0.81	0.44	0.98	0.48	0.87	0.8	<0.05	0.22	0.28

NM denotes that a measurement was not made in that sampling period. ED denotes erroneous data such as negative GP or R values. “Pairs” represents values of only coupled GP, NP and R measurements, i.e. when R and the corresponding GP and NP measurements are available for that same year. Linear regression slopes of metabolism vs. year (µg CL^−1^day^−1^), *r*^2^ correlations and *P*-values included at the bottom of the table.

**Fig.4.**
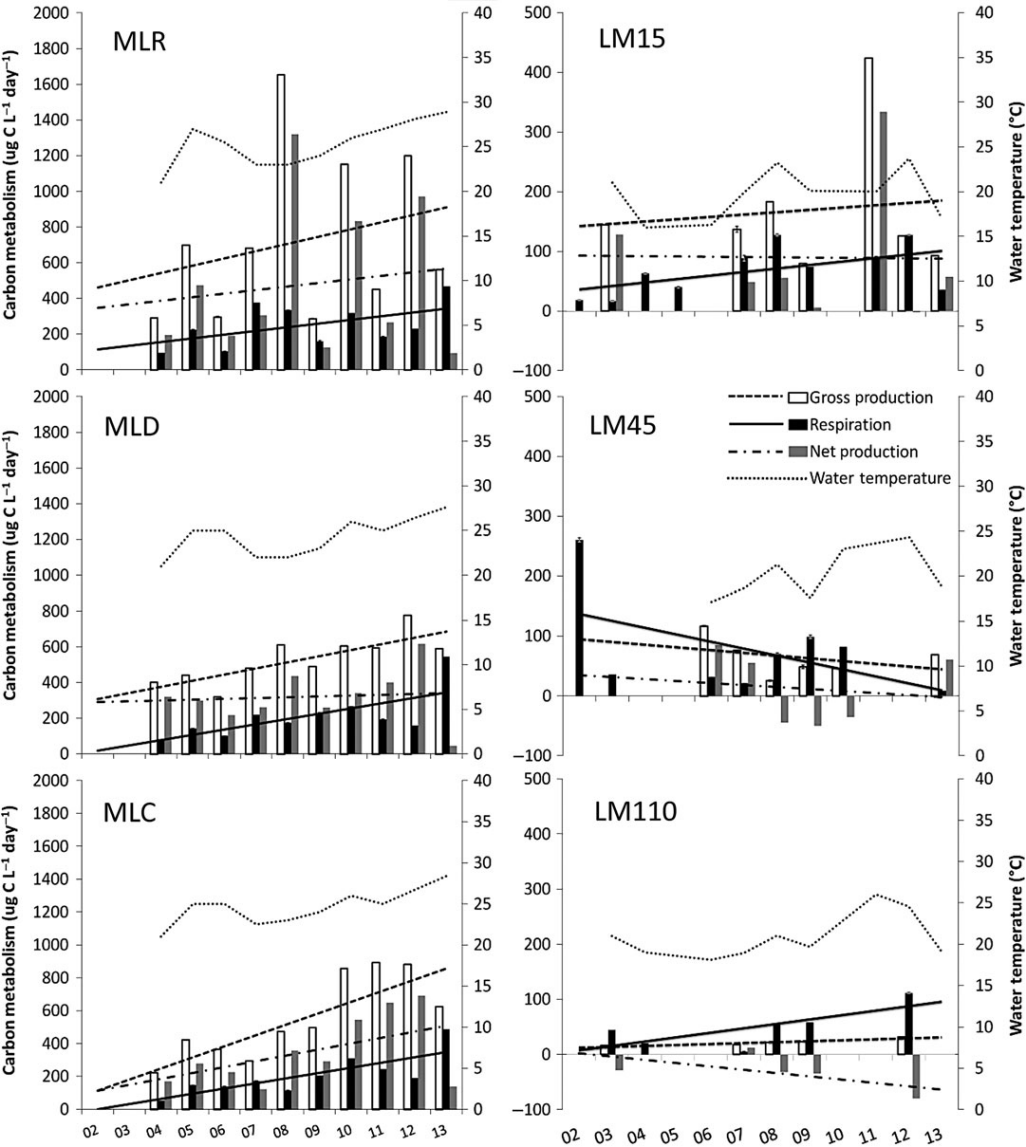
GP, R, NP and ambient surface water temperature measurements at MLR, MLD, MLC, LM15, LM45 and LM110. Each panel shows linear trend lines (see Table I for associated *r*^2^ values). The line graph shows how the water temperature throughout the years has changed at each of the sampling sites relative to concurrently measured metabolic rate processes.

Changes in NP along the transect during each year are shown in Fig.3. This shows a decrease in NP when each year is considered individually. Yearly variation and trends for each site individually are shown in Table I and Fig.4 for GP, R and NP. Although there is much variation over the 11-year period, the majority of the sites appear to show a gradual increasing trend for both GP and R, except for LM45. ML appears to have faster increase in GP and R over the period, while LM appears to have slower rates of increase for LM15 and LM110 (Fig.4). In a few cases, it appears that GP may be increasing at a slightly faster rate than R, as seems to be the case with MLR and MLC. The trends for LM45 are unique, in that there appears to be a decreasing trend for GP and R, but a cyclic pattern emerges where GP and R change dominance from 2006 to 2013 (Fig.4). Among all the sites, MLR, LM15 and LM45 showed the most variation of GP and R over the period as indicated by the lower *R*^2^ values (Table I). The rates of GP, R and NP were all significantly different from one another at each site based on a comparison of the slopes of the regressions (*P*-value < 0.05), except in the case of GP vs. NP at LM45 (*P*-value = 0.08).

Surface NP was much higher in ML than LM, with MLD and LM15 showing a consistent positive NP, LM45 showing a cyclical pattern of positive and negative NP and LM110 showing a consistent negative NP (Table I, Fig.4). NP is the highest in MLR with values reaching 1319 µg C L^−1^ day^−1^ and appears to show an increasing trend. In MLD and MLC, NP has increased steadily over the study period with less variation. The MLR and LM15 sites showed the most variation in NP.

We performed several analyses of other parameters in relation to GP, R and NP in an attempt to determine why there were differences in the calculated values of GP, R and NP. The five parameters we looked at were chl *a* concentration, surface water temperature, total precipitation, river discharge and daily average PAR, parameters that are likely to have the largest impact on the rates of GP, R and NP.

For the few data points available from LM and ML, averages of chl *a,* GP, R and NP all showed significant differences between ML and LM (Table II). This indicates a clear difference in trophic status between the two lakes. When metabolism values are plotted against the available chl *a* data, ML was most highly correlated to R (*r*^2^ = 0.85) and LM was most highly correlated to GP and R (*r*^2^ = 0.94 and *r*^2^ = 0.91, respectively). When the data from the two lakes are combined R was most correlated (*r*^2^ = 0.83) to changes in chl *a* (Table III).

**Table II:** The relationship of GP, R and NP to chl a in surface waters

	Muskegon Lake	Lake Michigan	*t*-test *P*-value
Average Chlorophyll *a* (μg L^−1^)	9.7	0.7	0.000002
Average GP (μg C L^−1^ day^−1^)	730	81	0.00002
Average R (μg C L^−1^ day^−1^)	300	24	0.001
Average NP (μg C L^−1^ day^−1^)	430	35	0.01

The averages of GP and R are representative of only values when both GP and R are coupled to chlorophyll *a*. A *t*-test was done to find if the two sites were different with respect to GP, R, NP and Chl *a*. All tests except average GP, R and NP per Chl *a* show a significant difference between Muskegon Lake and Lake Michigan.

**Table III:** Slope (m), linear correlation r^2^ and P-values for planktonic GP, R and NP values vs. chl a concentration separated into Muskegon Lake, Lake Michigan and combined data for the two zones (Muskegon Lake n = 9, Lake Michigan n = 3, All Sites n = 12)

	Slope			*r*^2^			*P*-value		
	Muskegon Lake	Lake Michigan	All Sites	Muskegon Lake	Lake Michigan	All Sites	Muskegon Lake	Lake Michigan	All Sites
GP (μg C L^−1^ day^−1^)	−25.60	139.70	83.80	0.07 (9)	0.94 (3)	0.47 (12)	0.51	0.16	<0.05
R (μg C L^−1^ day^−1^)	61.00	201.70	36.50	0.85 (9)	0.91 (3)	0.83 (12)	<0.05	0.41	<0.05
NP (μg C L^−1^ day^−1^)	−86.60	43.50	19.30	0.39 (9)	0.64 (3)	0.03 (12)	0.07	0.19	0.40

**Fig.5.**
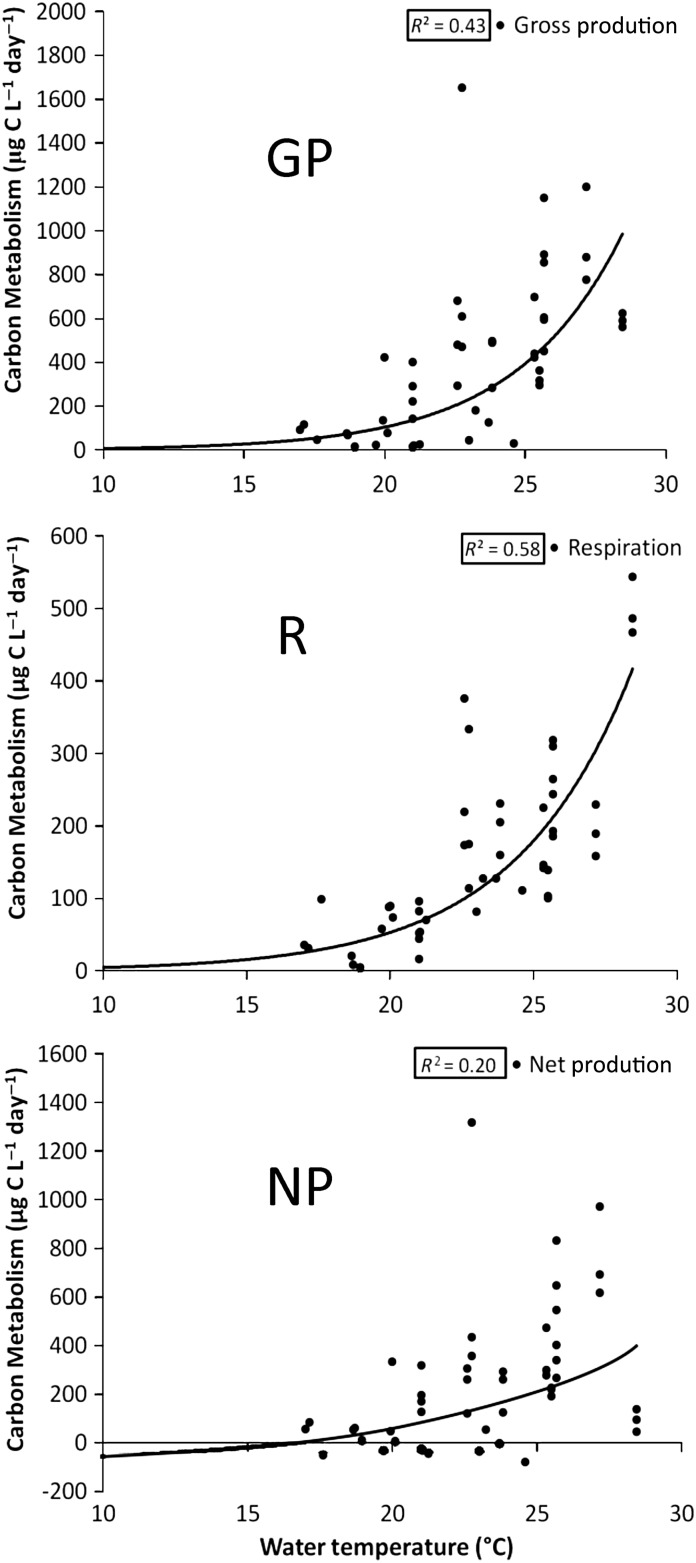
The relationship of planktonic GP, *R* and NP to ambient water temperature. All three show an exponential increase in metabolism as temperature increases, as indicated by exponential best-fit trend lines and associated *r*^2^ values.

Surface water temperature in ML showed an increasing trend since 2004, but was not highly correlated to GP, R and NP rates. However, as surface water temperatures have warmed at all ML sites, rates of GP, R and NP also noticeably increased (Fig.4). When plotted against temperature for all sites combined, there was a weak positive correlation for GP (*r*^2^ = 0.43) and R (*r*^2^ = 0.58) (Fig.5), and GP, R and NP were all significantly related to water temperature (*P*-values < 0.05).

GPP and R showed no significant trends associated with total precipitation for the 3 months before sampling, daily average of PAR, or discharge from Croton Dam (75 km upstream) either 15–90 days preceding or 15–60 days after sampling occurred. Given the lack of correlation, *r*^2^ values for total precipitation, river discharge and daily average PAR are not included.

## DISCUSSION

### Land-to-lake pelagic metabolism gradient

The overall pattern observed was a systematic decrease in GP, R and NP in the pelagic waters along the land-to-lake gradient, which has some far reaching implications for our understanding of the carbon cycle in land-margin ecosystems. Along this land-to-lake gradient, GP decreases at a faster rate compared with R. The surface NP trend suggests that sites closer to land are net autotrophic (carbon sinks), and offshore is net heterotrophic (carbon sources). For example, future models can be based on such measurements that would be able to predict zones of high primary and secondary production, and zones of net carbon sink or source to the atmosphere. The present study's average GP:R ratios are within the range for other lakes as determined by Hoellein *et al*. (Hoellein *et al*., 2013), even though most of the sites in the former study were heterotrophic compared with most of the sites in the present study that were autotrophic. The higher levels of GP and R in near-shore waters (e.g. LM15 to LM45) and inland lakes are expected with correspondingly higher inorganic nutrient and organic matter input. Previous research in the coastal zones of Lakes Michigan and Superior have also found similar decreasing rates of plankton metabolism in conjunction with decreasing concentrations of inorganic nutrients, DOC and chl *a* (Biddanda and Cotner, 2002; Urban *et al*., 2005). However, it is difficult to explain the observed tendency for offshore areas far from terrigenous sources to be net heterotrophic (Urban *et al*., 2005), unless we evoke significant episodic cross-shelf transport of carbon and nutrients in LM as demonstrated by more recent studies (Johengen *et al*., 2008; Kerfoot *et al*., 2008).

The yearly decrease of NP from land to lake was not always as systematic as the average trend indicates (e.g. Fig.3 compared with Fig.2). When we examined each site's yearly NP, a year of relatively more NP in the ML sites may not necessarily equate to more NP in the LM sites. This is shown by comparing 2011–2013. In 2011, NP actually increases across ML from MR to MC, and LM15 also shows its highest NP value during the course of the study. 2012 shows a fairly consistent decrease in NP across ML, and the LM sites LM15 and LM110 show their lowest NP values over the course of the study. However, in 2013, all ML Sites recorded some of their lowest values, but this was one of the highest NP years for LM15 and LM45. Thus, while the general trend is that NP decreases along the land-to-lake gradient, the rate of the decrease may be quite variable. Due to the dynamic circulation patterns in LM, exported NP from ML could be carried to other distant areas by way of the normally prevalent northward flow of currents along the eastern boundary of the lake, not necessarily fueling metabolism in the nearby LM study sites (Johengen *et al*., 2008; Kerfoot *et al*., 2008; McCormick *et al*., 2002). Others have observed that such northward and offshore hydrologic circulation is reflected in the distribution of plankton in LM (Carrick *et al*., 2001; Höök *et al*., 2006).

The average values of GP and R in the present study are well within values determined in other studies in the same and nearby lakes. In 2003, surface GP and R were measured in Mona Lake, a eutrophic drowned river-mouth lake 4 km to the south of ML (Biddanda *et al*., 2008). The Mona Lake GP rate was 1370 µg C L^−1^ day^−1^, significantly higher than the GP rate of 402 µg C L^−1^ day^−1^ for ML sites in 2004 (2003, data not available). Mona Lake R rate was 520 µg C L^−1^ day^−1^ compared with ML's 83 µg C L^−1^ day^−1^. Since ML is mesotrophic, higher GP and R rates would be expected in the eutrophic Mona Lake.

In the summer of 2010, another study used the same Muskegon Lake (MLD) and LM45 sample sites. In this study, separate surface water samples were collected at various points around the sample site and carbon metabolism rates were determined on each sample collected. As such, this method encompassed more spatial variance at these two sites, and showed MLD GP was 424 ± 5 µg C L^−1^ day^−1^ and *R* was 183 ± 7 µg C L^−1^ day^−1^ (D. Dila, personal communication), which were lower than in the present study for 2010 where GP and R at MLD were 605 ± 2 and 265 ± 2 µg C L^−1^ day^−1^, respectively. Both studies show a GP:R ratio of 2.3 in that year for ML. The previous 2010 study showed LM45 GP was 49 ± 3 µg C L^−1^ day^−1^ and R was 50 ± 3 µg C L^−1^ day^−1^ (D. Dila personal communication), compared with the GP and R from this study of 46 and 82 µg C L^−1^ day^−1^, respectively, reported in the present study (Table I). Variation observed between the two studies may be more related to different water masses on the sample days or PAR or temperature during bottle incubations. Surface NP values measured at M45 in the present study suggest that on average it is barely net productive.

The rates of production at LM110 in this study are much less than the rates seen in Fahnenstiel and Carrick (Fahnenstiel and Carrick, 1988). Their study indicates that offshore LM was net productive (35 µg C L^−1^ day^−1^) in 1988 (before the dreissenid mussel invasion), but the present study's earliest measurements in 2003 (post mussel invasion) puts LM110 NP at −28 µg C L^−1^ day^−1^, with net respiration. Respiration also increased from 12 µg C L^−1^ day^−1^ in 1988 (Fahnenstiel and Carrick, 1988) to 45 µg C L^−1^ day^−1^ in 2003 (present study). If this trend of relative enhancement of R (relative to GP) over time is real (although the origins of the additional substrate required to support this enhanced R is unknown), then it offers a new line of evidence for ongoing oligotrophication in this Great Lake environment. Overall, the GP:R ratios for ML and LM (LM15, LM45 and LM110) were within the range of previously measured GP:R ratios of lakes around the world with varying trophic levels (Duarte and Agusti, 1998).

### Carbon balance debate

How there can be more R than GP in oligotrophic waters similar to those of the Great Lakes and the open ocean has been widely debated (de Giorgio *et al*., 1997; Karl *et al*., 2003; Urban *et al*., 2005). Oligotrophic waters have been found to have a higher amount of bacterial heterotrophs relative to autotrophs (Biddanda *et al*., 2001). The role of bacterial heterotrophs in carbon flux is much higher in oligotrophic environments compared with eutrophic systems where phytoplankton dominates (Cotner and Biddanda, 2002).

The ML drowned river-mouth is the first body of water to receive nutrients from the Muskegon River. The organisms that inhabit the lake have the opportunity to actively assimilate and respire these nutrients and carbon, before it flows into LM. Typically, waters closer to land are more productive (e.g. higher nutrient cycling, higher autotrophy) and consequently tend to fuel higher rates of R, as long as watershed runoff does not lack limiting nutrients (Justić *et al*., 1995; Jickells, 1998). Marko *et al*. (Marko *et al*., 2013) performed a study that showed how ML serves as a trap for nutrients draining to LM. The amount of nutrients in ML is much higher than in LM, which suggests a high level of processing within ML so that many of those nutrients do not make it into LM (Defore, 2013; Marko *et al*., 2013).

Altogether this means that as water moves further from land, it becomes more heterotrophic. In other words, the ratio of GP to R gets closer to and in some cases goes below 1 into net heterotrophy, as seen in LM110. In the present study, the pelagic waters of drowned river-mouth lakes and near-shore areas were net carbon sinks, while starting at ∼9 km offshore (e.g. LM45), LM was carbon neutral or even a net source of carbon. This has also been seen in other oceanic and limnological gradient studies (del Giorgio *et al*., 1997; Kirchman, 1997; Urban *et al*., 2005).

It is easy to point to the metabolism of allochthonous carbon entering via rivers, but that may only be relevant to near-shore regions (<5 km from shore) before the nutrients become diluted (Urban *et al*., 2005). The M45 and M110 sites are 9 and 19 km offshore, respectively, which means something else must be feeding the system. Many studies have shown that these systems of oceans and offshore Great Lakes are supported largely through autochthonous GP (del Giorgio and Duarte, 2002; Scavia and Laird, 1987; Urban *et al*., 2005). This study sampled surface water during the summer, at a time after the spring bloom. In the summer of some years at LM45 and most years at LM110, the higher rates of R relative to GP are possibly fueled by the NP of the springtime when the bacteria are temperature limited (Scavia and Laird, 1987; Scavia *et al*., 1986). Thus, it is possible that the high points of GP and *R* do not coincide with one another.

The rapid decrease of pelagic GP compared with R is the major factor leading to the change in GP:R, NP and trophic status (Figs 2 and 3). A previous study in the Atlantic Ocean spanning across upwelling and oligotrophic waters has found that changing GP is the major controlling factor in the transition of a system from net autotrophy to heterotrophy (Arístegui and Harrison, 2002). These authors also found that R was far less variable than GP from autotrophic coastal areas to oligotrophic mid ocean areas. However, in our study, *R* was more variable and appeared to increase over time at our LM110 site (Fig.4). Such dynamics may be system specific, and, as in our pelagic system, changing over time. Our data show that LM, at 19 km offshore (LM110), has consistently been net heterotrophic, except for 2007, and is increasingly so due to increasing R over time. Thus, it will be critical to study the variability in GP than R in order to understand why a system is autotrophic or heterotrophic.

However, the notion of the prevalence of net heterotrophy in the oligotrophic oceans and Great Lakes has been questioned (Depew *et al*., 2006; Karl *et al*., 2003). One of the issues with calculating the carbon metabolism of a water body is sampling on appropriate temporal and spatial basis (Van de Bogert *et al*., 2012). Sampling needs to be done throughout the year and in many different areas of a lake to obtain a complete picture. Karl *et al*. (Karl *et al*., 2003) argued that sparse sampling of the North Pacific could lead to conclusions that it is heterotrophic, the system would be slightly net autotrophic if all of the autotrophic production during episodic blooms were considered which could offset the less variable heterotrophy throughout the year.

Excess production common in smaller lakes can settle to the bottom. If stratification sets up in a lake, decomposers can bring the hypolimnion of the lake into hypoxia or even anoxia, where anaerobic decomposers can release greenhouse gases such as nitrous oxide and methane (Rabalais *et al*., 2009). As the zone of hypoxia in the hypolimnion expands, fish are condensed in the epilimnion or shallows putting them at risk from predation (Rabalais *et al*., 2009). Time-series observations from the MLO have now revealed the annual recurrence of hypolimnetic hypoxia during the summer–autumn period in ML over the past 3 years (Biddanda *et al*., in prep.), suggesting that determining metabolism of water at several different depths at the sample site is important to determine its overall trophic status. The metabolic rates of this study, however, are only representative of the surface layer.

Our data and other studies give indications of increasing oligotrophication and the tendency of offshore LM waters to be heterotrophic, future analysis of ecosystem change in this lake would benefit from data with greater spatial and temporal resolution. Modern time-series sensors and sensor platforms are needed to monitor key carbon metabolism parameters in open water systems. Furthermore, development of working models of water-column metabolism based on time-series changes in dissolved oxygen at multiple depths could help characterize the temporal dynamics of autotrophy and heterotrophy (McNair *et al*., 2013). Such observations can greatly advance our knowledge of ecosystem metabolism over that offered by bottle incubation methods.

### Intense near-shore carbon cycling

Over the last decade, we have seen a pattern of consistently increased GP, R and NP at MLD and MLC sampling sites, and highly variable GP, R and NP at the MLR site. The MLR sampling site seems to be much more influenced by watershed runoff as its metabolism is much more variable than MLD or MLC (Fig.4). Years of extremely high GP and NP may indicate a more nutrient rich runoff from the watershed. Indeed, pulses of nutrients from agricultural catchments have been found to cause high levels of productivity in the receiving oligotrophic basins such as the Gulf of California (Beman *et al*., 2005). Similarly, the MLR is much more variable, being the first area to receive high concentrations of nutrients and carbon from the watershed. The nutrients and organic matter are then partially used up and diluted by the time they reach the MLD and MLC sites on the other side of the lake, making them less variable.

**Fig.6.**
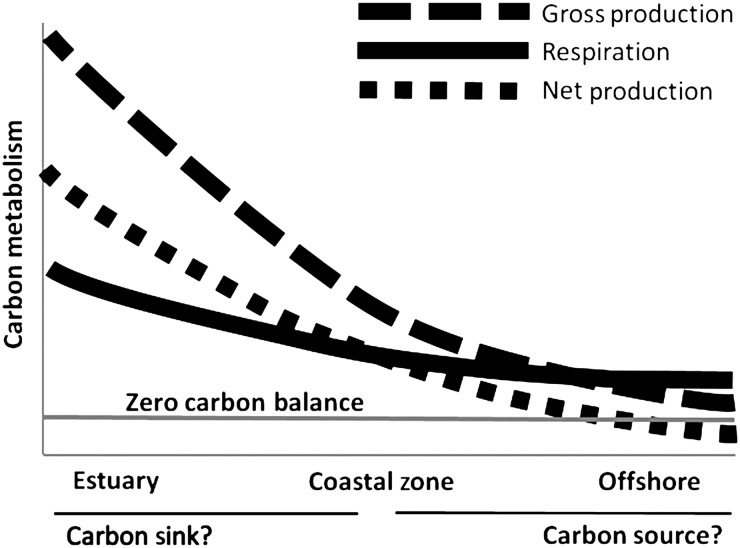
Generalized conceptual diagram of systematic variability in planktonic gross production, respiration and net production along the land-to-water gradient in aquatic ecosystems based on the present study. Both the axes scales are relative, and the grey horizontal line near the bottom serves as the “zero carbon balance” reference line.

At LM15, there is a lot of variation in GP, R and NP (Fig.4). Such variations possibly stem from changing flow patterns and composition of the outflow from rivers emptying into LM further south (Biddanda and Cotner, 2002). Currents along the southeastern coast of LM typically flow North (Beletsky *et al*., 1999), bringing with them the diluted contents of the Grand River plume (entering LM ∼18 km south of Muskegon River) into the study transect off of Muskegon. Thus, observed oscillations in GP, R and NP in the LM transect may reflect the influence of the Grand River watershed as well as water flow patterns within LM, in addition to the regionally significant Muskegon River plume. Such coastal zones on shelf of LM may support higher levels of NP in the future, as continental shelves tend to become increasingly autotrophic due to anthropogenic activities (Bauer *et al*., 2013).

### Reduced autotrophy due to invasive mussels at the nearshore-offshore transition zone

At LM45 from 2006 to 2010 (Fig.4), the consistent pattern of decreasing GP with a concurrent rise in R may be due to invasive mussels (Evans *et al*., 2011; Fahnenstiel *et al*, 2010). Dreissenids have an extraordinary ability to filter the water column, especially when they are in the unprecedented numbers that can be found in the Great Lakes (Hecky *et al*., 2004; Pothoven and Fahnenstiel, 2013). Vanderploeg *et al*. (Vanderploeg *et al*., 2010) found that the highest abundance of dreissenid quagga mussels (*Dreissena bugensis*) was located between 30 and 50 m in LM. Their research at the 30–50 m isobath also showed that the biomass of quagga mussels increased exponentially from 1998 to 2008, as did the amount of the water column filtered every day (Vanderploeg *et al*., 2010). At LM45, the exponential increase in quagga mussels in recent years is positively correlated to an increase in R and inversely correlated to the decrease in GP for LM45 from 2006 to 2010 and may have led to a shift in overall trophic status. During 2006 and 2007, LM45 had a positive NP, but in 2008–2010 LM45 shifted to negative NP (Fig.4). This indicates a shift from autotrophy to heterotrophy, influencing this sites ability to be a net sink or source of carbon to the atmosphere. Unlike the Arístegui and Harrison (Arístegui and Harrison, 2002) study which showed that trophic status was affected by mainly GP, in the present study, the shift in trophic status was likely caused by changes in both GP and R.

However, Mida *et al*. (Mida *et al*., 2010) suggest that mussels of the genus *Dreissena* may not have access to the surface water during summer stratification. Other possibilities for the pattern we observed are that LM45 may be impacted by the near-shore phosphorus shunt created by filtering dreissenids, thus cutting off a large amount of nutrients that would otherwise disperse out to M45 (Hecky *et al*., 2004). Another possible explanation is that the counter-clockwise rotation pattern of the southern basin currents of LM combines with the relatively steep drop-off where LM45 is located creating a higher amount of upwelling despite stratification (Kerfoot *et al*., 2008). Such enhanced regional upwelling may in turn lessen the intensity of summer stratification enabling mussels to more efficiently filter the water column. The phytoplankton at the surface may be coming in contact with hypolimnetic waters that have been filtered of their nutrients by rising mussel populations. The observed pattern of decreased GP from 2006 to 2010 at LM45 (Fig.4) suggests that dreissenid mussels are still able to affect the surface GP even during stratified periods.

This pattern was altered in 2013 when the measured amount of GP compared with that of 2007 at the same site is much higher. There are a couple of possibilities that could be the cause of such a dramatic recent increase in GP. The first is perhaps another evidence for a change in flow patterns in LM. LM45 may have received an influx of nutrients from another source due to a change in surface flow or a mixing event (Johengen *et al*., 2008). Another possibility relates to the population dynamics of the dreissenid mussels. Nalepa *et al*. (Nalepa *et al*., 2009) found that in some areas of the Great Lakes, the dreissenid populations increased rapidly then declined, which is common among dreissenid populations (Strayer and Malcom, 2006). A similar population decline event could now be happening with the quagga mussels in the 30–50 m depth isobath. In the 2-year gap in this study between 2010 and 2013 measurements, the population of quagga mussels may have declined drastically leading to an increase in productivity at LM45.

### Steady state of offshore surface waters

The rates of GP at LM110 have stayed fairly constant over the course of the study (Fig.4). This is consistent with other research in Southern LM at similar depths. In offshore areas more than 100 m depth, no significant decline in summer chl *a* concentrations (typically July–September) was found since the mussel invasion beginning in the 1980s up to 2007 and 2008 (Fahnenstiel *et al*., 2010; Mida *et al*., 2010). More recently, Pothoven and Fahnenstiel (Pothoven and Fahnenstiel, 2013) determined that July chl *a* concentrations were significantly higher between the years 1995 and 2000 compared with 2007–2011, but September did not differ. Estimates at LM110 in mid-July to early-August indicate that between 2003 and 2013, there has not been any significant change in productivity at LM110. In relation to the Pothoven and Fahnenstiel (Pothoven and Fahnenstiel, 2013) study, perhaps the mid-summer measurements in this study are more reflective of late summer (September), than early summer (July). The lack of changes in productivity at M110 could be because there are negligible amounts of dreissenid mussels to filter the water column at more than 90 m depth along the transect (Vanderploeg *et al*., 2010). Furthermore, mussels filter at the bottom and may not have access to the surface waters during summer stratification (Mida *et al*., 2010) in a non-upwelling zone.

Increased R at LM110 over the 11-year monitoring period may be due to changing dynamics of the bacterial population in the increasingly oligotrophic waters. Bacteria are known to dominate in oligotrophic waters, so it is possible that they become more prolific as the populations of summer phytoplankton decrease (Biddanda *et al*., 2001; Pothoven and Fahnenstiel, 2013). The bacteria may be becoming more efficient with their resources as well, needing to depend on decreasing amounts of phytoplankton every year or switching potential food sources. Approximately 80% of the DOC pool in previously studied oligotrophic lakes is allochthonous in origin, yet bacteria prefer to consume the DOC of autochthonous origin (Bade, 2004; Kritzberg *et al*., 2004). Bacteria must be rapidly consuming the fresh algal DOC and then metabolizing the ever-present terrestrial DOC (Guillemette *et al*., 2013; Kerfoot *et al*., 2008). Offshore respiration could be fueled by relatively refractory terrigenous DOC which is able to move great distances and fuel metabolism in areas where autochthonous labile DOC has already been consumed (Guillemette *et al*., 2013; McCallister and del Giorgio, 2008). Despite the nearly constant levels of low GP at M110, the site's NP has decreased, making this offshore site increasingly heterotrophic. This observation contrasts with the previously held view that trophic status is determined more by GP than R (Arístegui and Harrison, 2002). While this site has been consistently heterotrophic, it is becoming more so, primarily due to increasing R.

### Drivers of pelagic metabolism along the land-to-lake gradient

#### Phytoplankton and Chl a

The amount of phytoplankton in the samples must affect the amount of GP and R directly. ML was eutrophic, and LM was oligotrophic, as indicated by significantly different chl *a* values and metabolic rates. We did find a moderately positive correlation of GP to chl *a* and a strongly positive correlation of R to chl *a* when all the data were combined from both sites. ML's lack of correlation of GP with chl *a* may be a product of higher nutrient concentrations controlling GP more so than overall chl *a*. However, in LM, which is nutrient poor, GP and R may be regulated more by overall chl *a* concentration. On the whole, R has the highest correlation, while NP has the lowest correlation to chl *a*. The NP trend agrees with previous findings; however, the R trend does not. Increasing R and NP have been found to have a low correlation with increasing chl *a*, explaining why NP did not show any significant difference but was inconsistent with the higher correlation of R to chl *a* (Arístegui and Harrison, 2002). Unfortunately, due to a limited data set for chl *a* in ML and LM, the trends were difficult to resolve with certainty in the present study.

#### Temperature

When GP and R were plotted against temperature for all sites, the analysis revealed that GP and R both have a moderately positive correlation to water temperature. Increasing metabolism in ML, specifically, was accompanied by correspondingly increasing temperature through the years, whereas LM sites individually showed few signs of any influence from temperature. The correlation between the growth rate of phytoplankton and temperature has been observed earlier for both GP and R. Eppley (Eppley, 1972) introduced “The Eppley Curve” which summarized the finding that as temperature increases so does the growth rate of phytoplankton. It also shows how the growth of different species of phytoplankton increases with temperature at different rates leading to the obscuring of the increase when all the species are measured together (Fig.5). In the PROTECH model used by Elliott *et al*. (Elliott *et al*., 2006), as temperature increases, algal biomass increases similarly. Furthermore, higher rates of bacterial GP and R are seen with increasing temperature (Pomeroy *et al*., 1991; Rabalais *et al*., 2009; Scavia and Laird, 1987). While ML has shown trends of both increasing GP and R, GP seems to be increasing faster than R. However, a study in marine mesocosms has shown that GP:R decreased with increasing temperature (Wohlers *et al*., 2009). This discrepancy with the present study may be due to ongoing changes in regional land-use and precipitation pattern over the years in the Muskegon River Basin (Marko *et al*., 2013). Increased regional precipitation and resulting land-runoff may be leading to increasing GP and R in ML. Increased terrestrial inorganic nutrient input (and not correspondingly high organic matter input) into the Lake may override the different effects of temperature on GP and R, causing GP to increase faster than R, whereas the effect of temperature alone might show the opposite response.

#### Precipitation and discharge as a proxy for phosphorous loading

In freshwater ecosystems, large watershed size to the lake surface area ratio correlates positively to phosphorous concentration and metabolism (Biddanda and Cotner, 2002; Kalff, 2002). Specifically in lakes and drowned river-mouths, phosphorous is the main driver of metabolism and chlorophyll *a* concentration (Beisner *et al*., 2003; Hoellein *et al*., 2013). Increased temperatures and rainfall have been linked to increased phosphorous loadings and eutrophication (Jeppesen *et al*., 2009).

We analyzed the total amount of precipitation and discharge to see if it could account for the varying GP and R, and act as a proxy for fluxes in phosphorous concentration. There was no significant linear correlation when we plotted GP, GP: precipitation, R, and R: precipitation against precipitation. There were also no significant correlations between total discharge from the Croton Dam and any preceding interval or lagged interval. Thus, precipitation and river discharge may not necessarily be a representative proxy of the total nutrients, such as phosphorous, entering the system.

#### Photosynthetically active radiation

Our analysis revealed that the amount of GP in the bottles was independent of the amount of PAR received during the incubation period. The GP at each of the sites showed a different pattern relative to one another in consideration of the amount of light available for photosynthesis. On some days when PAR was relatively higher, GP would be lower than on days when there was lower PAR. Previous studies have shown that the color of the water may limit light penetration and therefore limit photosynthesis more than nutrient concentration (Karlsson *et al*., 2009). However, if we consider efficiency as carbon production per unit PAR, the colored water of ML still has a photosynthetic efficiency that is higher than out in the relatively clear waters of LM. Therefore, it is conceivable that nutrient concentration plays a larger role than light-limitation in shaping changes in pelagic GP and R in this system.

### Significance of systematic variability in GP, R and NP along the land-to-lake gradient

In the present study, pelagic autotrophic and heterotrophic processes involved in ecosystem metabolism change distinctly and systematically from the drowned river-mouth waters to offshore oligotrophic waters of the ocean and Great Lakes. Understanding how these ecosystems differ among their potential GP, R and NP ranges helps us define drivers of metabolism in each ecosystem (Hoellein *et al*., 2013). ML and near-shore LM support high levels of carbon cycling aided by autochthonous GP and allochthonous inputs from the watershed. Based on their high NP values, these near-shore systems may be actively storing carbon in the sediment and fueling secondary production. Any sinking NP could also very well be respired in the hypolimnion making the bottom waters net heterotrophic. Offshore LM surface metabolism is supported by minimal levels of autochthonous GP and any residual allochthonous subsidies, and are therefore, likely to function as net sources of carbon as indicated by LM110′s negative NP value. As the amount of autochthonous GP changes from land to lake, the ratio of allochthonous carbon vs. autochthonus carbon respired by bacteria may increase further into LM (McCallister and del Giorgio, 2008). Much of the nutrients in oligotrophic waters are in a dissolved organic form that heterotrophic bacteria are better at acquiring than phytoplankton (Cotner and Biddanda, 2002).

The patterns of land-to-lake gradients in production and respiration observed in the present study may extend to other coastal ecosystems of the world regulating the carbon cycle (Fig.6). Perhaps the levels of GP and R in other systems may show similar relationships to the ML/LM system, and fit into a general pattern such as that found in the present study. Furthermore, now that studies have demonstrated that inland waters are important within the global carbon cycle (Cole *et al*., 2007; Raymond *et al*., 2013; Tranvik *et al*., 2009), future measurements of metabolism should be carried out throughout the year and along both directions from the land's edge, including shallow headwater streams, offshore pelagic ecosystems and involve the water column as well as the benthos.

The carbon cycle of coastal ecosystems is a dynamic component of the global carbon cycle (Schlesinger and Berhardt, 2013). As the human population and standard of living continue to increase, agricultural runoff from the world's continents is fueling enhanced phytoplankton production, and likely increased respiration as well, in coastal waters globally (Jickells, 1998; Justić *et al*., 1995; Beman *et al*., 2005). There is mounting evidence that agriculture and urbanization-driven eutrophication is currently decreasing carbon dioxide evasion while increasing carbon sequestration in inland lakes (Pacheco *et al*., 2013). Recent observations suggesting that the coastal ocean carbon cycle may have indeed shifted from its traditional role as a source of atmospheric carbon dioxide in pre-industrial period to an active sink for carbon dioxide in more modern times (Bauer *et al*., 2013) further emphasizes the need for such cross-ecosystem and long-term process measurements in the world's coastal zones.

## FUNDING

This research was supported by a NASA Michigan Space Grant Consortium Seed Grant (GVSU-426581), an EPA Great Lakes Restoration Initiative Grant (GLOOE00460-0) a Michigan State University Water Initiative Research Grant (OSTR-GR100020) to B.A.B, and a Community Foundation for Muskegon County grant to Dr. Alan Steinman (GVSU). Funding to pay the Open Access publication charges for this article was provided by the Grand Valley State University libraries and the Center for Scholarly and Creative Excellence.
